# The Prognostic Value of Inflammatory Indexes in Patients With Severe Traumatic Brain Injury

**DOI:** 10.1002/brb3.70711

**Published:** 2025-08-21

**Authors:** Yongxiang Yang, Dongbo Zou, Chen Yin, Xiansong Zhu, Yunxing Li, Yuan Ma, Jingmin Cheng

**Affiliations:** ^1^ Department of Neurosurgery The General Hospital of Western Theater Command Chengdu China; ^2^ Department of Gerontology Sichuan Lansheng Brain Hospital Chengdu China; ^3^ College of Medicine Southwest Jiaotong University Chengdu China; ^4^ The Affiliated Hospital of Southwest Medical University Luzhou China

**Keywords:** clinical outcome, GOS, inflammatory index, severe traumatic brain injury

## Abstract

**Objective:**

This study aims to investigate whether the inflammatory indexes including neutrophil to lymphocyte ratio (NLR), platelet to lymphocyte ratio (PLR), lymphocyte to monocyte ratio (LMR), and systemic inflammatory indexes (SIIs) can prognosis the outcome of patients with severe traumatic brain injury (sTBI).

**Methods:**

A total of 118 sTBI patients were retrospectively recruited from June 2015 to June 2024. First, the clinical data including baseline clinical features, hematological indexes at 1/3/7 days after hospital admission, were collected, and the Glasgow Coma Scale (GCS) at 3 months after discharge was recorded. Then, the absolute value of SII, NLR, PLR and LMR was calculated, and the prognostic value of them was further analyzed via comparative, relevant, and regression statistical methods.

**Results:**

Compared to sTBI patients in the favorable outcome group, the absolute value of white blood cell (WBC) and neutrophil at 1/3/7 days, and the absolute value of monocyte at 1 day and NLR at 3 days after admission was higher in sTBI patients of the unfavorable outcome group. ROC curve and multivariate logistic regression analysis indicated that NLR at 3 days after admission was an independent prognostic factor for GOS in patients with sTBI.

**Conclusions:**

NLR at 3 days after admission might be a new, accurate, and objective inflammatory index, which had prognostic value in the clinical outcome prediction for patients with sTBI.

## Graphical abstract

NLR at 3 days after brain injury might be a new, accurate, and objective inflammatory index, which had prognostic value in the clinical outcome prediction for patients with sTBI.

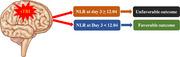

## 1 Background

Traumatic brain injury (TBI) is a common neurosurgical disease caused by external mechanical forces, which has increasingly incidence and is a major cause of death and disability worldwide (Maas et al. 2022). TBI is a complex and dynamic pathological process including primary injury and secondary injury. Primary injury refers to the destruction of brain tissue due to focal intracranial hemorrhage, epidural hematoma, subdural hematoma, cerebral contusion, and diffuse axonal injury, primarily determining the outcome of the TBI patient at the time of injury (Mishra et al. 2022). Secondary injury is triggered by the initial brain injury, mainly referring to oxidative stress, ischemia, edema, and inflammatory responses (Shi et al. 2019). Clinical treatment methods for the management of primary injury such as traditional decompressive craniectomy is far more adequate for the prognosis improvement of TBI patients (L. Chen et al. 2022). Recent studies have demonstrated that the clinical outcome of TBI can be predicted by secondary inflammatory indexes, and might be improved by preventing potentially modifiable secondary injuries such as inflammatory responses (Mishra et al. 2022). In particular, newly studies have shown that systemic inflammation responses are associated with poor outcomes in patients with severe TBI (sTBI), and systemic inflammatory indexes (SIIs) might provide reliable prediction results for physicians to perform clinical interventions more effectively and improve the prognosis of sTBI (Mishra et al. 2022). Herein, it is of great significant to study whether SII can prognosis the outcome of sTBI patients dynamically.

Inflammatory responses after TBI are triggered by the presence of injured neural cells and release of injury‐related inflammatory molecules, which is featured by the activation of resident cells, migration and recruitment of peripheral neutrophils, and release of inflammatory mediators (Yuxiong et al. 2022). Among these inflammatory responses, neutrophils are the most important and abundant cells that immediately respond and play key roles in the acute phase and post‐injury period after TBI (L. Chen et al. 2022; Ozeren 2022). Circulating neutrophils in peripheral blood begin to respond within a few hours of trauma, whose absolute number is doubled at 3–4.5 h and reaches to the peak within 12–24 h after TBI (Alam et al. 2020). Those activated neutrophils further migrate to the brain and release various inflammatory cytokines and factors, which in turn recruit additional blood‐borne neutrophils and monocytes into the injured brain and propagate the inflammatory cascade (Alam et al. 2020). Recent studies have demonstrated that some inflammatory biomarkers such as the neutrophil to lymphocyte ratio (NLR), platelet to lymphocyte ratio (PLR), lymphocyte to monocyte ratio (LMR), and SII had the potential to predict functional outcome after sTBI (L. Chen et al. 2022; Ozeren 2022; Sabouri et al. 2020; Y. Chen et al. 2021; Kadir and Ayca 2024). Most of these research reported that NLR at Day 1 after admission could be a prognostic indicator of outcome after sTBI (L. Chen et al. 2022; Ozeren 2022; Sabouri et al. 2020; Y. Chen et al. 2021; Kadir and Ayca 2024), while Chen et al. found the peak NLR at Days2–4 after admission had better predictive value (Kadir and Ayca 2024). Moreover, the release of inflammatory biomarkers after sTBI is associated with blood‐brain barrier (BBB) permeability, which had been verified to reach first peak within few hours after injury persisting for 3–4 days, and reach a second peak at 5 days (Kadir and Ayca 2024). Taking together, it is meaningful to conduct research to investigate the prognostic value of NLR, PLR, LMR, and SII in the outcome prediction at 1/3/7 days after injury, as the inflammatory response after sTBI is a dynamic process.

As far as we know, no study have investigated the dynamic change of inflammatory biomarkers including NLR, PLR, LMR, and SII at different time points and their prognostic value in the outcome prediction after sTBI. Herein, the present study was conducted to analyze the differences of NLR, PLR, LMR, and SII between sTBI patients with good outcome and bad outcome. Moreover, the prognostic value in outcome prediction of these parameters was explored using univariate and multivariate regression analysis. The aim of this study was to clarify the prognostic value of inflammatory biomarkers and provide reliable prediction results for physicians to perform clinical interventions more effectively and improve the prognosis of patients with sTBI.

## 2 Materials and Methods

### 2.1 Setting

This retrospective study was conducted in China at The General Hospital of Western Theater Command in Chengdu, which has separate intensive care unit (ICU) beds for TBI patients. The ICU has professional intensivist‐led teams consisting of critical care physician and nurse, who are in charge of 24‐h medical care. Ethics Committee of the Faculty of The General Hospital of Western Theater Command in Chengdu gave permission for this research. All the studying process in this research was carried out in accordance with the approved guidelines.

### 2.2 Patients

sTBI was defined based on International Classification of Diseases (ICD 9/10 codes) for TBI, as well as Glasgow Coma Score (GCS) ≤ 8, ventilatory assistance for more than 24 consecutive hours, and intracranial pressure (ICP) monitoring device. All eligible sTBI patients who were treated in The General Hospital of Western Theater Command in Chengdu from June 2015 to June 2024 were included, and the sample size was not determined. The clinical data including complete admission and hospitalization records of sTBI patients were collected by experienced neurosurgeons. The inclusion criteria include the following: (1) age ≥ 18years; (2) discharge diagnosis of sTBI (ICD 9/10 codes); (3) types of injuries in patients with sTBI: primary TBI caused by car accidents, high fall injury, and external object strikes; (4) admission to the hospital within 12 h after injury, and completion of basic laboratory tests for blood indicators in the emergency department; and (5) GCS ≤ 8 at admission. The exclusion criteria include the following: (1) the admission and hospitalization information was incomplete; (2) presence of extracranial injury (such as orthopedic/chest/cardiac/abdominal/pelvis and so on); (3) preexisting cardiac disease (such as myocardial ischemia/infarction, arrhythmia, heart failure); (4) combined with liver/renal/lung failure, hematological disease, infection disease, malignancy, and pregnancy; (5) death within the first 12 h after hospitalization; and (6) admission to our hospital after emergency surgery in other hospitals.

### 2.3 Clinical Care of Patients With sTBI

Once patients arrived at the emergency department, standard treatments and management were carried out immediately according to the Brain Trauma Foundation Guidelines (Volovici et al. 2019). According to the Glasgow Coma Scale (GCS), patients with GCS ≤ 8 at admission were categorized as sTBI (Schucht et al. 2025). All the patients received comprehensive neurological evaluation and underwent cranial CT scan subsequently. Repeat CT scan was conducted whenever patients presented the indication of clinical deterioration or the sign of ICP elevation. Other routine clinical examinations including chest x‐ray, abdomen ultrasound, electrocardiogram, and laboratory tests including hematology, urine and feces analysis, lipid and coagulation profile, and multiorgan (cardiac, liver, renal) function analysis were conducted within 3 h after hospitalization. The ICU protocols and treatments mainly include vital signs monitoring, medical pharmacotherapy, and surgical treatment according to the latest Brain Trauma Foundation guidelines (Volovici et al. 2019).

### 2.4 Data Collection

The collected data included clinical features, radiologic findings, hematology profile, treatment method, and final outcome. Clinical features involved baseline demography, vital signs, time before admission, key symptoms, and major diagnosis. According to radiological findings on CT, sTBI was defined as DAI (diffuse axonal injury), CE (cerebral edema), ICH (intracerebral hemorrhage), and S‐EH/SAH (subdural‐epidural hematoma/subarachnoid hemorrhage). Hematology profile included the total count of platelet (PLT), neutrophils, lymphocyte, and monocyte at 1/3/7 days after hospital admission. And the value of SII (platelet × neutrophil/lymphocyte), NLR (neutrophil/lymphocyte), PLR (platelet/lymphocyte), and LMR (lymphocyte/ monocyte) was calculated based on those counts. Treatment methods mainly involved medical pharmacotherapy and surgical treatments such as the evacuation of hematomas and decompressive craniectomy. Clinical outcomes included hospital length of stay (LOS), 30‐day mortality, and Glasgow Outcome Scale (GOS) at 3 months collected via telephone, outpatient, and other follow‐up methods. GOS was classified as death, persistent vegetative state, severe disability, moderate disability, and low disability. Accordingly, sTBI patients were divided into the favorable outcome group (moderate and low disability) and the unfavorable outcome group (death, persistent vegetative state, and severe disability).

### 2.5 Data Analysis

Data distribution was determined using the Kolmogorov–Smirnov test. Measurement variables were expressed as mean values ± standard deviations (M ± SD). Differences between two groups were analyzed by Unpaired *t* test with Welch's correction for parametric data and Wilcoxon single‐rank test and Mann–Whitney *U* test for nonparametric data. Categorical variables were compared using chi‐square test, Fisher's exact test, and Yates’ correction. Receiver operating characteristics (ROC) curve analysis test was used to assess the predictive value of variables and the “Youden index” was used to determine the cutoff value. Multivariate logistic regression analyses were used to investigate the prognostic value of SIIs. SPSS version 18.0 software (SPSS Inc., USA) and GraphPad Prism 8 were used to perform the analysis, and two‐tailed *p* < 0.05 was considered statistically significant. Photoshop software (Adobe Software, Inc., USA) was used to draw the figure.

## 3 Results

### 3.1 Clinical Features of the Recruited Patients With sTBI

**FIGURE 1:**
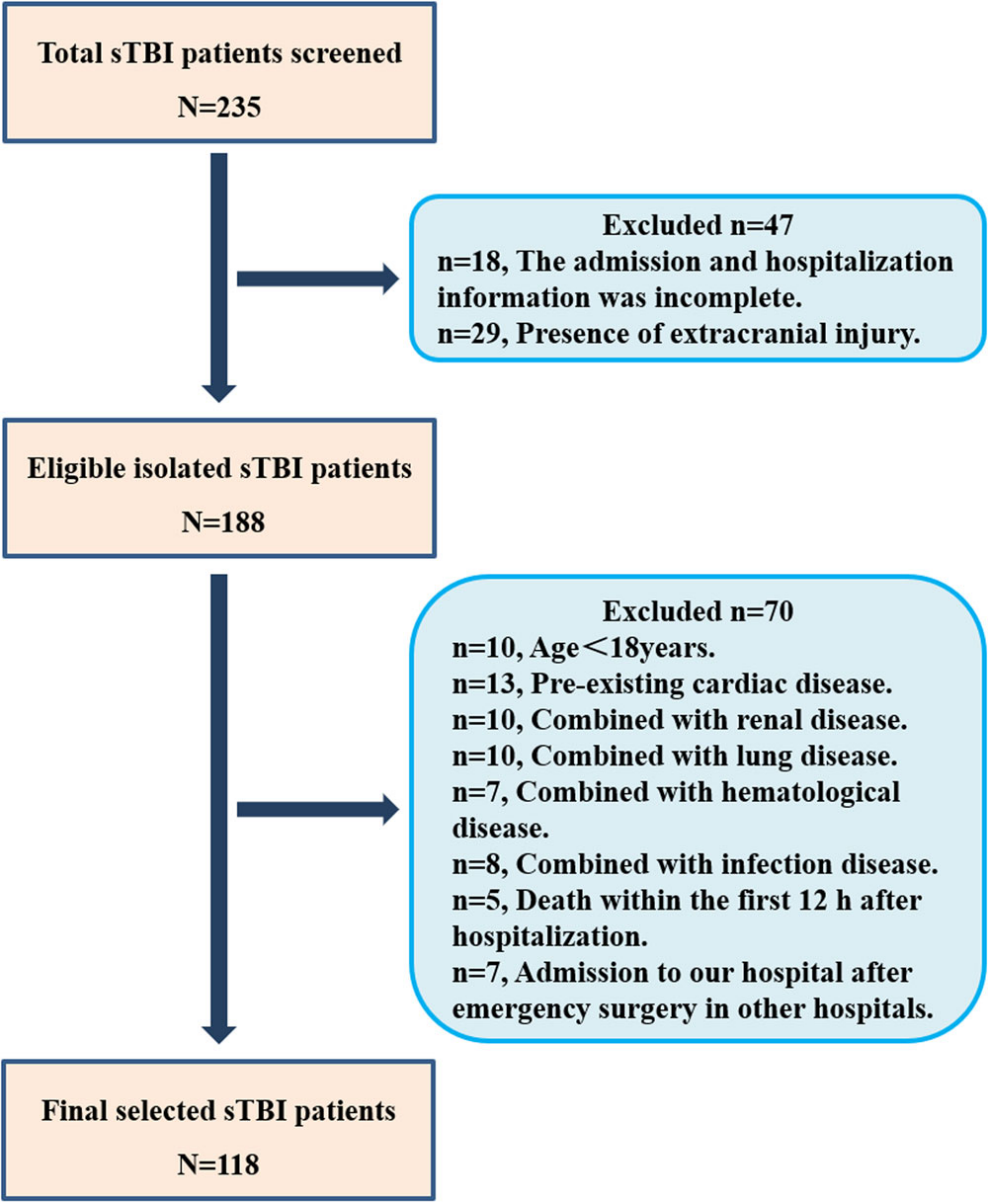
The selection flowchart of patients with sTBI.

At last, 118 sTBI patients were recruited according to the inclusion and exclusion criteria as described in Section 2. The selection flowchart was illustrated in Figure 1, and clinical features of the recruited patients with sTBI are shown in Table 1. The mean age was 52.1 (±15.3) years, the number of male patients was more than female patients (77.9% vs.22.1%). The median GCS score of patients on admission was 5 (IQR: 3–8). According to CT findings, 81.4% of sTBI patients had cerebral hemorrhage (12.5% were EH, 36.5% were SH, 33.3% were SAH, and 17.7% were ICH), 11.0% of patients had a DAI and 7.6% of patients had a CE. According to the Marshall classifications, 66.9% of sTBI patients had brain stem compression, and 72.8% of sTBI patients had midline shift. As for the clinical outcome evaluated by GOS at 3 months after injury, 13.6% of patients died, 5.1% were in a persistent vegetative state, 30.5% had severe disability, 24.5% had moderate disability, and 26.3% had low disability. Average hospital LOS was 32.1days, and 35.6% of patients had a LOS longer than 30 days. According to GOS score, 60 (50.8%) and 58 (49.2%) patients were classified into the favorable outcome group and the unfavorable outcome group, respectively.

**TABLE 1:** Clinical characteristics of patients with sTBI.

**Variable**	**Value**
Gender (*n*, %)	
Male	92 (77.9%)
Female	26 (22.1%)
Age (mean ± SD, years)	52.1 ± 15.3
GCS (median［IQR］)	5［3–8］
Major CT diagnosis (*n*, %)	
Intracranial hemorrhage	96 (81.4%)
Subdural hemorrhage	35 (36.5%)
Subarachnoid hemorrhage	32 (33.3%)
Intracerebral hemorrhage	17 (17.7%)
Epidural hematoma	12 (12.5%)
Diffuse axonal injury	13 (11.0%)
Cerebral edema	9 (7.6%)
Marshall classifications of CT (*n*, %)	
Brain stem compression	79 (66.9%)
Midline shift	86 (72.8%)
GOS at 3 months	
1‐Death	16 (13.6%)
2‐Persistent vegetative state	6 (5.1%)
3‐Severe disability	36 (30.5%)
4‐Moderate disability	29 (24.5%)
5‐Low disability	31 (26.3%)
Average hospital LOS (days)	32.1 ± 34.5
Hospital LOS ≥ 30 days (*n*, %)	42 (35.6%)
Clinical outcome (*n*, %)	
Favorable	60 (50.8%)
Unfavorable	58 (49.2%)

Abbreviations: GCS, Glasgow Coma Scale; GOS, Glasgow Outcome Score; LOS, length of stay.

### 3.2 Differences of Inflammatory Indexes Between the Favorable and Unfavorable Outcome Groups

**FIGURE 2:**
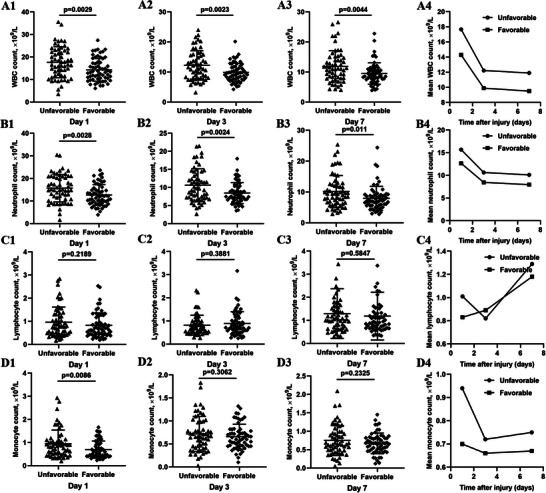
The comparisons of the value and alteration trends of WBC, neutrophil, lymphocyte, and monocyte between the favorable and unfavorable outcome groups in patients with sTBI at 1/3/7 days. *p *< 0.05. WBC, white blood cell.

**FIGURE 3:**
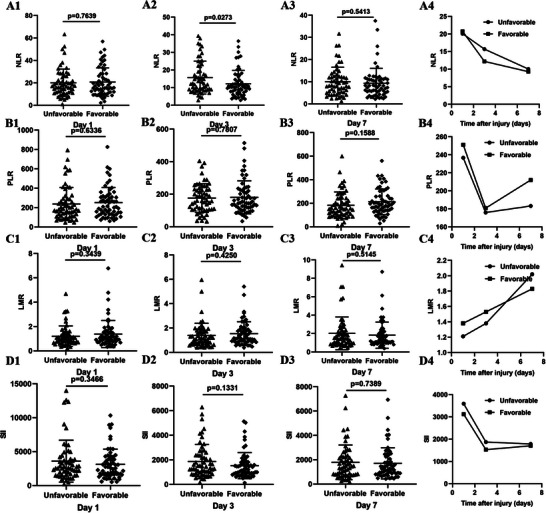
The comparisons of the value and alteration trends of NLR, PLR, LMR, and SII between the favorable and unfavorable outcome groups in patients with sTBI at 1/3/7 days. NLR, neutrophil to lymphocyte ratio; PLR, platelet to lymphocyte ratio; LMR, lymphocyte to monocyte ratio; SII, systemic inflammatory indexes. *p*<0.05.

The comparison of inflammatory indexes between the favorable and unfavorable outcome groups was illustrated in Figures 2 and 3. Compared to patients with sTBI in the favorable outcome group, the absolute value of white blood cell (WBC) and neutrophil at 1/3/7 days, and the absolute value of monocyte at 1 day and NLR at 3 days after admission was higher in sTBI patients of the unfavorable outcome group (all *p *< 0.05). The analysis on the trends of these inflammatory indexes over time indicated that the absolute value of WBC, neutrophil, and NLR declined gradually, and the value of LMR raised gradually from Day 1 to Day 7 in sTBI patients of the favorable and unfavorable groups. And, the value of lymphocyte, monocyte, PLR, and SII decreased first and increased subsequently from Day 1 to Day 7 in sTBI patients of the favorable and unfavorable outcome groups. Moreover, it is worth noting that the descent degree of monocyte at 3 days was obviously higher in the unfavorable outcome group than in the favorable outcome group.

### 3.3 Correlations Between Inflammatory Indexes and Clinical Outcomes of Patients With sTBI

**FIGURE 4:**
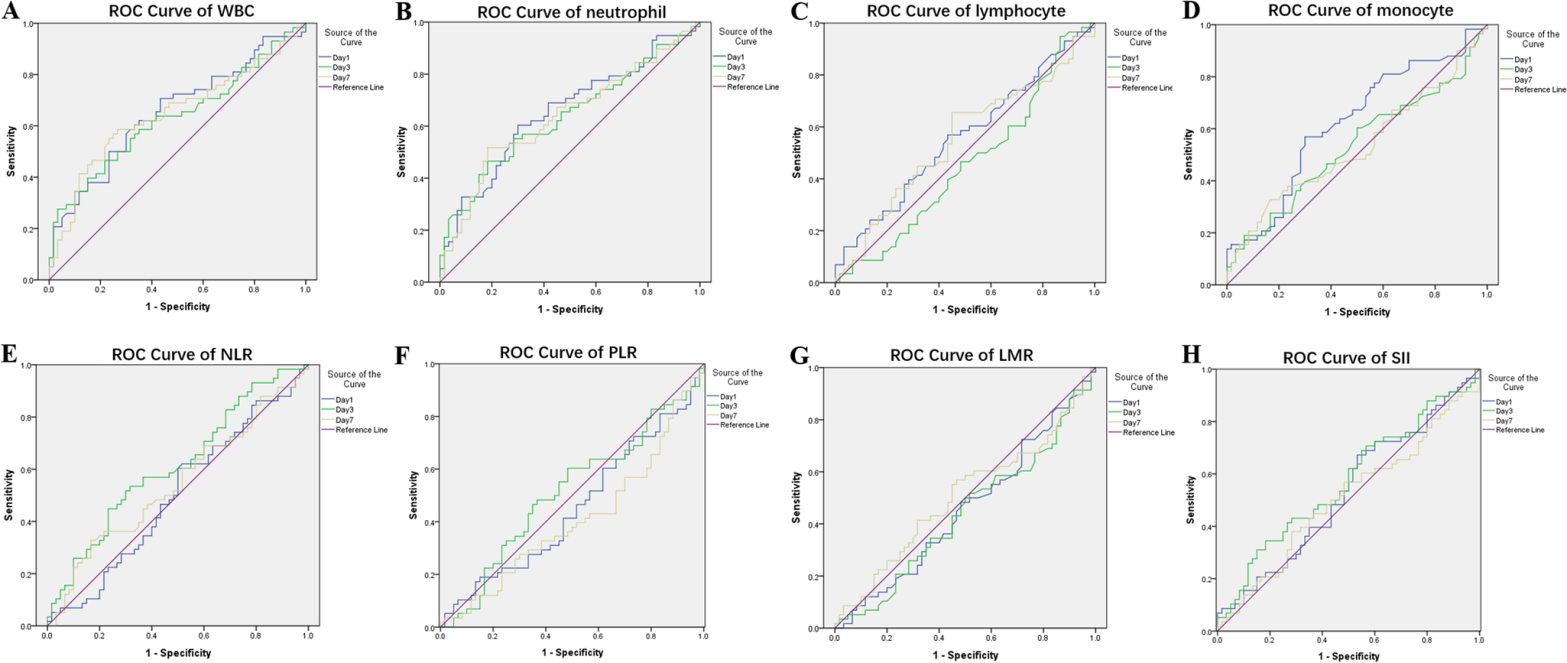
ROC curve of WBC, neutrophil, lymphocyte, monocyte, NLR, PLR, LMR, SII, and GOS in patients with sTBI at 1/3/7 days after hospital admission.

**FIGURE 5:**
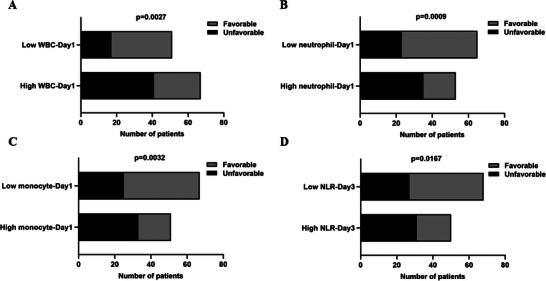
Chi‐square test of correlations between WBC, neutrophil, and monocyte at Day 1; NLR at Day 3; and clinical outcome of patients with sTBI. *p* < 0.05.

In order to find out which inflammatory indexes could influence the clinical outcomes of patients with sTBI preliminarily, ROC curve analysis and chi‐square test were performed. ROC curve analysis indicated that the GOS of sTBI patients might be influenced by the value of WBC, neutrophil, and monocyte at 1 day, and the value of NLR at 3 days after admission (Figure 4). The cutoff value of WBC at Day1 was 14.075 × 10^9^/L (AUC = 0.652, *p* = 0.004), neutrophil at Day1 was 14.5 × 10^9^/L (AUC = 0.653, *p* = 0.004), monocyte at Day1 was 0.755 × 10^9^/L (AUC = 0.622, *p* = 0.022), and NLR at Day3 was 12.04 (AUC = 0.611, *p* = 0.037). According to the cutoff value, patients with sTBI were divided into high WBC and low WBC group, high neutrophil and low neutrophil group, high monocyte and low monocyte group, and high NLR and low NLR group. Furthermore, correlations between these four inflammatory indexes and clinical outcome of patients with sTBI were analyzed by chi‐square test. Results indicated that the value of WBC at Day1, neutrophil at Day1, monocyte at Day1, and NLR at Day3 was correlated with the clinical outcome of patients with sTBI (Figure 5).

### 3.4 The Prognostic Value of Inflammatory Indexes in Patients With sTBI

Above results indicated that the value of WBC, neutrophil, and monocyte at Day1 and NLR at Day3 was correlated with the clinical outcome of patients with sTBI. Aiming to deeply analyze the prognostic value of inflammatory indexes in patients with sTBI, multivariate logistic regression analysis was performed subsequently. Results indicated that the value of NLR at 3 days after admission (OR = 2.419, 95% CI: 1.091–5.360, *p* = 0.030) was an independent prognostic factor for GOS in patients with sTBI (Table 2).

**TABLE 2:** Multivariate logistic regression analysis on the inflammatory indexes and GOS.

**Variable**	**OR (95% CI)**	***p* value**
WBC—Day 1	1.203 (0.335–4.317)	0.776
Neutrophil—Day 1	2.373 (0.646–8.713)	0.193
Monocyte—Day 1	1.625 (0.623–4.240)	0.321
NRL—Day 3	2.419 (1.091–5.360)	0.030^*^

Abbreviations: CI, confidence interval; NLR, neutrophil to lymphocyte ratio; WBC, white blood cell.

^*^ *p* < 0.05.

## 4 Discussion

As far as we know, this is the first study that investigated the prognostic value of inflammatory indexes including PLR, NLR, LMR, and SII in clinical outcome of patients with sTBI dynamically. Main findings were as follows: (1) sTBI patients in the unfavorable outcome group had higher level of WBC and neutrophil at 1/3/7 days, monocyte at 1 day and NLR at 3 days after hospital admission. (2) The GOS of sTBI patients might be influenced by the value of WBC, neutrophil, and monocyte at 1 day and NLR at 3 days after hospital admission. (3) NLR at 3 days after hospital admission was an independent prognostic factor for GOS in patients with sTBI. These results indicated that NLR had better predictive value than other inflammatory indexes, which might be an effective predictor for outcome prognosis in patients with sTBI.

TBI has two successional pathological stages, one is the primary damage featured by neural tissue injury caused by primary force, another one is the secondary damage induced by the inflammatory response to brain injury (Ozeren 2022). Inflammatory response after TBI has recently become a study hotspot, which is characterized by the activation of resident cells in the brain, the migration and recruitment of inflammatory cells in the peripheral blood, and the release of inflammatory mediators (Yuxiong et al. 2022). Pro‐inflammatory cytokines are released from damaged neuronal cell within a few hours of brain trauma, thus leading to the activation of neuroimmune cells in the brain such as microglia immediately (Shao et al. 2022). In turn, activated microglia are polarized to the pro‐inflammatory type, thereby leading to more intense inflammatory responses and more secretion of inflammatory cytokines in brain tissue (Shao et al. 2022). Further, inflammatory cytokines cause the degradation of tight junction, cytoskeletal rearrangement, inappropriate activation of endothelial cells, and impairment of BBB integrity (Schmitt et al. 2023). The disruption of BBB permeability leads to the leukocyte infiltration and the recruitment of neutrophils to the site of injury in the first hour after trauma (van Hameren et al. 2024; Mason et al. 2021). The upregulation of pro‐inflammatory cytokines and activation of neutrophils further stimulate leukocyte and monocyte, acting an essential role in the activation of endothelial cell and platelet, leading to secondary damage after TBI eventually (Siwicka‐Gieroba et al. 2019). The interactions between neuroimmune cells in the brain and inflammatory cells in the peripheral blood provide us a window to study the neuroinflammation and secondary brain damage after sTBI. Moreover, recent studies have demonstrated that inflammatory indexes on admission might have the potential to predict the clinical outcome of sTBI patients, but their conclusions are inconsistent. In this study, we retrospectively investigated the prognostic value of several inflammatory indexes in clinical outcome prediction of patients with sTBI at 1/3/7 days after hospital admission.

In recent years, several studies have explored the prognostic roles of inflammatory indicators such as NLR, PLR, and LMR in predicting the clinical outcomes of patients with sTBI (L. Chen et al. 2022; Ozeren 2022; Kadir and Ayca 2024; Siwicka‐Gieroba et al. 2019; Thapa et al. 2021). Thereinto, NLR is the most frequently investigated one, which is a simple and reliable biomarker obtained from a complete blood test and is generally considered to be an indicator of inflammation before any clinical findings can be observed (Ozeren 2022). Some studies have shown that NLR included a simple hematological investigation in predicting the mortality and GOS outcomes of sTBI (Kadir and Ayca 2024; Siwicka‐Gieroba et al. 2019; Thapa et al. 2021; Corbett et al. 2019), while other studies have indicated that the predictive performance of NLR was poorer than other predictive biomarkers (Bilgi et al. 2021). Moreover, W. Chen et al. (2018) have demonstrated that NLR might serve as a readily available clinical marker for preoperative prognosis in patients with sTBI, and high NLR on admission is associated with poor prognosis. However, another study showed that NLR at admission was not an effective predictor for the outcome of sTBI (Dolmans et al. 2020). As for other inflammatory indicators such as PLR, LMR, and SII, a few recent research have examined their ability to predict the outcomes of sTBI. One recent study has shown that SII had better predictive performance than NLR, PLR, and LMR, and SII could be a new, accurate, and objective clinical predictor to improve the accuracy of GOS prediction in patients with sTBI at 6 months after discharge (L. Chen et al. 2022). Our study found that sTBI patients in the unfavorable outcome group had higher level of WBC and neutrophil at 1/3/7 days, monocyte at 1 day, and NLR at 3 days after hospital admission than in the favorable outcome group, indicating the potential prognostic value of these four indicators. Further, we investigated the prognostic value of WBC and neutrophil at 1/3/7 days, monocyte at 1 day, and NLR at 3 days after hospital admission, which is a dynamic manner for the first time. ROC curve analysis indicated that the GOS of sTBI patients might be influenced by the value of WBC, neutrophil, and monocyte at 1 day and NLR at 3 days after admission. Multivariate logistic regression analysis further revealed that NLR at 3 days after admission was an independent prognostic factor for GOS in sTBI patients. Similarly, one previous study has shown that NLR is a dynamic entity in patients with sTBI, starting to rise at 1 day and reaching to the peak at 2–4 days after brain injury (J. Chen et al. 2019; Kadir and Ayca 2024). This study also found that the peak value of NLR was a better prognostic indicator for outcome prediction in patients with sTBI, compared to NLR at Day 1 (J. Chen et al. 2019; Kadir and Ayca 2024). The possible explanation for this phenomenon might be the fact that the first peak of increased BBB permeability is observed within the first few hours after injury and persists for 3–4 days, and a second peak may occur after 5 days after brain injury as a result of microglial activation (Siwicka‐Gieroba et al. 2019).

In conclusion, we retrospectively investigated the prognostic value of SII, NLR, PLR, and LMR in the outcome prediction of patients with sTBI in a dynamical manner. We found sTBI patients in the unfavorable outcome group had higher level of WBC and neutrophil at 1/3/7 days, monocyte at 1 day, and NLR at 3 days after hospital admission. GOS of sTBI patients might be influenced by the value of WBC, neutrophil, and monocyte at 1 day and NLR at 3 days after hospital admission, and NLR at 3 days after hospital admission was an independent prognostic factor for GOS in patients with sTBI. In all, NLR at 3 days after hospital admission might be a new, accurate, and objective inflammatory index that can prognosis the clinical outcome of patients with sTBI.

## Author Contributions

**Yongxiang Yang**: writing–review and editing, writing–original draft, investigation, data curation, supervision, methodology. **Dongbo Zou**: software, investigation, data curation, methodology. **Chen Yin**: methodology, software, data curation, investigation. **Xiansong Zhu**: validation, data curation. **Yunxing Li**: investigation, software. **Yuan Ma**: writing–review and editing, methodology, supervision, validation. **Jingmin Cheng**: methodology, writing–review and editing, validation, supervision.

## Conflicts of Interest

The authors declare no conflicts of interest.

## Peer Review

The peer review history for this article is available at https://publons.com/publon/10.1002/brb3.70711

## Data Availability

The data that support the findings of this study are available from the corresponding author upon reasonable request.
